# Clinical analysis of intraoperative radiotherapy during breast-conserving surgery of early breast cancer in the Chinese Han population

**DOI:** 10.18632/oncotarget.5716

**Published:** 2015-10-17

**Authors:** Xin Wang, Jiaqi Liu, Wenyan Wang, Qinfu Feng, Xiang Wang

**Affiliations:** ^1^ Department of Breast Surgical Oncology, Cancer Hospital of Chinese Academy of Medical Sciences, Beijing, China; ^2^ Department of Radiotherapy, Cancer Hospital of Chinese Academy of Medical Sciences, Beijing, China

**Keywords:** breast cancer, breast-conserving surgery, intraoperative radiotherapy

## Abstract

**Purpose:**

While results of intraoperative radiotherapy (IORT) during breast-conserving surgery (BCS) have been reported when used either as a boost at the time of surgery or as the sole radiation treatment, the clinical safety and cosmetic outcome of IORT in the Chinese Han population has not. This report reviews oncologic and cosmetic outcomes for Chinese Han breast cancer patients who received IORT either as a boost or as their sole radiation treatment at our hospital.

**Method:**

From July 2008 to December 2012, 50 early-stage Chinese Han breast cancer patients received BCS and IORT, either as boost or as their sole radiation treatment. Patients received adjuvant chemotherapy or hormonal therapy, according to our institution's guidelines. Patients were followed to determine oncologic events, short-term toxicity and overall cosmesis.

**Results:**

With a median follow-up of 51.8 months (range 22.6 months to 75.7 months), 2 patients (4.0%) developed local relapses and were salvaged by mastectomy. There were no metastases and no deaths. The average wound healing time was 17 days. Three patients (6.0%) developed postoperative infection, 5 patients (10.0%) had delayed wound healing, and 2 patients (4.0%) experienced wound edema. There were no lyponecrosis or hematomas observed. The evaluation of cosmetic outcome showed 44 patients (88.0%) graded as excellent or good while 6 patients (12.0%) were graded as fair or poor. No patients experienced radiotherapy related acute hematological toxicity, but 3 patients (6.0%), all IORT boost patients, developed skin pigmentation.

**Conclusion:**

For early-stage breast cancer patients, intraoperative radiotherapy after breast-conserving surgery in the Chinese Han population is both safe and reliable and has resulted in very acceptable cosmetic outcomes.

## INTRODUCTION

Whole-breast external beam radiotherapy (EBRT) following breast-conserving surgery (BCS) has equivalent overall survival and local recurrence rates (LRR) compared with total mastectomy [1–3], and has the advantage of preserving the breast. The LRR after BCS and conventional radiotherapy (RT) is typically less than 1% per year, and ranges between 4–7% at 5 years [4, 5]. A further reduction in LRR of up to 40% can be achieved by an additional external radiation boost of 10–16 Gy to the tumor bed [6]. However, in many countries, up to 30% patients who undergo BCS for early breast cancer decline RT or even opt for mastectomy to avoid radiotherapy because they live a substantial distance from a RT center, or have significant co-morbidities, or difficulties that prevent them from attending daily treatment, especially true for older women [7, 8]. In addition, the increasing use of oncoplastic breast surgery which often rearranges the tumor bed position, amplifies the challenge of accurate delivery of RT boost to the tumor bed, even when the surgeon routinely places cavity clips to help in targeting the tumor bed [9, 10].

However, 5 to 7 weeks of RT after BCS, which is both inconvenient and costly to patients, might not always be needed. Schnitt et al. [11] and Veronesi et al. [12] indicate that the vast majority of recurrences after BCS occur in the index quadrant. It has been observed that up to 90% of local recurrences are at or near the original operation site [13–15], and the LRR in other quadrants was approximately 15%, approaching the risk of contralateral breast cancer [6]. This observation, and the desire to increase the acceptance of RT after BCS, has led to an interest in Accelerated Partial Breast Irradiation (APBI) techniques that focus radiation specifically on the index quadrant. Intraoperative radiotherapy (IORT) is one technique for delivering APBI. IORT delivers the radiation at the time of surgery, thus accurately defining the target volume of the breast and specifically targeting the tumor cavity.

Because IORT is delivered in a single fraction during surgery with the surgeon directly visualizing the target, it enables maximum precision in the delivery of the dose and reduces the dose to healthy surrounding tissues [16]. Compared with whole breast radiotherapy, it has the following advantages: 1) accurate positioning of radiation field, high surface dose, and uniform target dose; 2) single high-dose radiation potentially avoids residual tumor cell repair and proliferation that can occur due to delay in delivering fractionated radiotherapy, thereby improving the biological effect; 3) avoids dose to vital organs (heart, lungs, etc.); 4) less dose to normal tissues, reducing the risk of radiation-induced second primary tumors; 5) shortened overall treatment time; 6) better cosmetic outcome with less pigmentation, dryness and swelling of skin; 7) reduction in healthcare costs. The disadvantages of IORT are: 1) increased operating times; 2) lack of final pathological results before delivering IORT; 3) small increase local recurrence rates compared with conventional EBRT in two RCTs [17, 18]; 4) staff training and operating room equipment efforts; 5) expensive devices [19].

While IORT has been studied by a large number of clinical trials in America and Europe, the clinical efficacy and cosmetic outcome of IORT in Chinese Han population or even Asian people, still lack evidence. In our study, we enrolled 50 Chinese Han early breast cancer patients to receive breast-conserving surgery and IORT using a mobile electron accelerator (Mobetron 1000, IntraOp, Sunnyvale, CA). Postoperative recurrences, metastases, complications and cosmetic outcome were followed up to evaluate the safety and feasibility of IORT after BCS.

## MATERIALS AND METHODS

### Patients

Chinese Han patients with early breast cancer who were eligible for BCS were offered participation in an IORT study at the Cancer Hospital of the Chinese Academy of Medical Sciences, Beijing, China using a mobile electron accelerator (Mobetron^®^, Intraop Medical, Sunnyvale, CA, USA).

Preoperative mammography, ultrasound and MRI were used to exclude multifocal cancer, and preoperative imaging and clinical examination was used to exclude any obvious axillary lymph node involvement. Patients found on postoperative pathological examination to have more than 3 axillary lymph node metastases were eliminated from the study and treated with modified radical mastectomy and postoperative radiotherapy. This study was conducted with approval from the Ethics Committee of the Cancer Hospital Chinese Academy of Medical Sciences and in accordance with the declaration of Helsinki. Written informed consent was obtained from all participants.

From July 2008 to Dec 2012, 50 patients were entered into the study. Fourteen patients (28%) had IORT as a boost followed by 5 weeks of WBI, while 36 patients (72%) had IORT as their sole radiation treatment. The median age of the patients was 46.9 years (range 26–70 years). All tumors were ≤ 3 cm in size without invasion of the skin, adjacent muscles or chest wall. The detailed clinical data of patients is summarized in Table 1.

**Table 1:** Patient characteristics (*n* = 50)

Characteristics	IORT boost	IORT only	Total number	Percentage of total (%)
Patient Treatment	14	36	50	100
Age (years) ≤35	2	4	6	12
36–55	11	23	34	68
≥56	1	9	10	20
Tumor position				
upper outer quadrant	11	20	31	62
lower outer quadrant	1	5	6	12
upper inter quadrant	2	10	12	24
lower inter quadrant	0	1	1	2
Tumor maximum diameter				
≤2cm	11	29	40	80
>2cm	3	7	10	20
Histological Examination				
Ductal carcinoma *in situ*	1	3	4	8
Invasive ductal carcinoma	13	30	43	86
Mucinous adenocarcinoma	0	2	2	4
Intraductal papillary carcinoma	0	1	1	2
Lymph node metastasis (pathology)				
NO	9	33	42	84
N1	5	3	8	16
Immunohistochemistry				
ER+/and/or/PR+	9	24	33	66
ER- and PR-	5	12	17	34
Her-2+	6	3	9	18
HER-2−	8	33	41	82
Chemotherapy	9	24	33	66
Targeted therapy*	1	3	4	8

* Herceptin treatment for one year

### Surgical operation

Of the 50 patients, 27 underwent BCS and sentinel lymph node biopsy, 22 BCS and ipsilateral axillary lymph node dissection (ALND), and one was a resection of local recurrence after previous BCS. Technetium-99m was injected into four points along the side of the areola hypodermic 2 hours before surgery. A gamma detector was used to label sentinel lymph nodes. The axillary incision over the skin and subcutaneous tissue along the axillary fold was performed to remove and biopsy the radiolabeled sentinel lymph node. The ALND included clearing of level I and II lymph nodes in the Berg muscle-based categorization, which should be at least 10. Using a curved or radial incision, the skin and subcutaneous tissue were cut with the tumor at the center. The tumor and approximately 2 cm of adjacent normal breast tissue were resected and underwent frozen-section pathologic diagnosis to ensure the margins were clear. If the resection margins were positive, re-excision would be performed. Fortunately, there was no positive margin on final pathology in this study. A purse-string suture was applied to the gland around tumor bed and was tightly wrapped so that when the electron beam applicator was centered on the sutured gland, it would ensure homogeneity of the irradiated target. A 2 mm-thick lead disk was positioned under the breast gland to protect chest-wall and underlying ribs, lung and heart.

### IORT Treatment

IORT was delivered, either as a boost or APBI IORT treatment, using a mobile electron accelerator (Mobetron^®^, Intraop, USA). Prior to the surgery the Mobetron was placed in the operating room and disinfected. The radiation oncology team selected the proper electron beam energy and collimator diameter such that 90% of the prescribed dose would cover at least 1.5 cm of tissue adjacent to the margins and at least 1 cm of breast tissue on the chest wall surface. The energy of the electron beam ranged from 6 to 12 MeV and the prescription dose to the target area was 9 Gy (boost) or 20 Gy (APBI IORT) using single irradiation of 3–5 min duration.

### Postoperative treatment

All patients received post-operative adjuvant chemotherapy and/or endocrine therapy appropriate to their clinical staging and pathological findings. The 14 patients who received IORT as a boost, also received postoperative irradiation of 50 Gy to the whole breast (25 fractions × 2 Gy/fraction) using 6 MV x-rays. Postoperative radiation was delayed for 126 days (4 months) for those IORT boost patients requiring adjuvant chemotherapy.

### Follow-up and cosmetic outcome evaluation

Patients were followed every 3-months from the surgery by either outpatient visits or by telephone. The primary outcomes of the study were local recurrence, metastases and mortality. Secondary outcomes included complications and cosmetic evaluation and quality of life. The cosmetic outcome was scored as excellent/good/fair/poor in accordance with the grading system developed by Harris et al. [20] The treated breast was compared with the untreated breast to score these items. Excellent meant there was no difference between breasts; Good meant there was only a slight difference; Fair was when a marked difference was present; and Poor was in case of a significant difference. The scores given for each patient by different reviewers were combined into a single average.

## RESULTS

### Follow-up

The median follow-up was 51.8 months (range 22.6 to 75.7 months). We observed 2 (4.0%) recurrences, occurring 12 months and 25 months after surgery. Both were single fraction patients, one DCIS and the other a Mucinous tumor. Both patients were salvaged by total mastectomy. No metastases or deaths occurred. The overall outcome and adverse events of the enrolled patients are shown in Table 2.

**Table 2:** Overall outcome of the enrolled patients (*n* = 50)

Characteristics	Number	Percentage (%)
primary outcomes		
Local recurrences	2	4
Metastasis	None	
Death	None	
Cosmetic outcome		
Excellent or good	44	88
Fair or poor	6	12
Wound healing (average wound healing time was 17 days)		
Infection	3	6
Healing delay	5	10
Wound edema	2	4
Fat deliquescence	None	
Adverse events		
Skin pigmentation	3	6
Ipsilateral breast infection	None	
Acute hematological toxicity	None	
Haematoma	None	

### Wound healing

The average wound healing time in study group was 17 days. Infection occurred in 3 patients (6.0%), healing delay in 5 patients (10.0%), and wound edema in 2 patients (4.0%). However, there was no fat deliquescence or hematomas.

### Cosmetic evaluation of postoperative breast appearance

Cosmetic outcome was evaluated after surgery at 1 year. 44 patients (88.0%) were graded as excellent or good, while 6 patients (12.0%) were rated as fair or poor.

### Adverse events

In this study, 3 patients (6.0%) showed mild skin pigmentation. There was no intraoperative or postoperative radiation-related acute hematological toxicity or other adverse reactions.

## DISCUSSION

While BCS plus 5–7 weeks of RT in early stage breast cancer remains the standard treatment to preserve the breast in much of the world, both the Canadians [21] and the UK START-B studies [22–24] found that d 40 Gy in 15 fractions gave equivalent results. Consequently, 3 weeks of RT after BCS, with or without a boost, is rapidly becoming the standard treatment because the shortened treatment schedule is more convenient and less costly than the 5 week RT approach. In some countries, such as China, even 3–4 weeks of RT can be an obstacle for women living far from a radiation center or for older women who are less concerned with cosmesis. Our goal in this study, was to see if we could increase breast preservation in China by radically reducing the time for the required radiation. Veronesi et al. [25] and others [26] have estimated that a single fraction of 21 Gy is biologically equivalent the standard 5–7 weeks of RT.

Even though the recently published ELIOT trial [17] with mature follow-up of 5.7 years, resulted in a higher LRR than the EBRT group (4.4% vs. 0.4%), there importantly was no difference in survival even at 10 years. In addition, grade 3 or 4 skin complications were significantly reduced with IORT. It has been suggested that improved selection of patients might reduce the rate of local recurrence to < 2% at 5 years [26, 27] with IORT.

In our study, despite a much younger patient population and other higher risk factors than published in other studies, there were just 2 cases (4.0%) of LRR, but no metastases or deaths. The cosmesis and toxicities were quite acceptable, with 44 patients (88.0%) graded as excellent or good, and only 3 patients (6.0%) with wound infection 5 patients (10.0%) with healing delay, and 2 patients (4.0%) with wound edema. No patient had fat deliquescence or hematomas. Furthermore, unlike another Asian study [26], we experienced no hypertrophic scarring. The average wound healing time in our study group was 17 days. Through longer follow-up is needed, no patients experienced radiation injury, which indicates the clinical safety of IORT and encourages us to continue to evaluate and refine this treatment technique with the aim of increasing breast preservation in China without jeopardizing patient survival.

## CONCLUSION

For early-stage breast cancer patients, intraoperative radiotherapy after breast-conserving surgery in the Chinese Han population is both safe and reliable and has resulted in very acceptable cosmetic outcomes. IORT has the potential to greatly increase the breast preservation rate in China, though a longer follow-up will be needed to fully understand which patients can safely benefit from this treatment approach.

## Abbreviations

ANLD: axillary lymph node dissection
APBI: Accelerated Partial Breast Irradiation
BCS: breastconserving surgery
EBRT: external beam radiotherapy
IORT: intraoperative radiotherapy
LRR: local recurrence rates
RT: radiotherapy.

## References

[1] Veronesi U, Cascinelli N, Mariani L, Greco M, Saccozzi R, Luini A, Aguilar M, Marubini E (2002). Twenty-year follow-up of a randomized study comparing breast-conserving surgery with radical mastectomy for early breast cancer. N Engl J Med.

[2] Clarke M, Collins R, Darby S, Davies C, Elphinstone P, Evans E, Godwin J, Gray R, Hicks C, James S, MacKinnon E, McGale P, McHugh T (2005). Effects of radiotherapy and of differences in the extent of surgery for early breast cancer on local recurrence and 15-year survival: an overview of the randomised trials. Lancet.

[3] Fisher B, Anderson S, Bryant J, Margolese RG, Deutsch M, Fisher ER, Jeong JH, Wolmark N (2002). Twenty-year follow-up of a randomized trial comparing total mastectomy, lumpectomy, and lumpectomy plus irradiation for the treatment of invasive breast cancer. N Engl J Med.

[4] Huston TL, Simmons RM (2005). Locally recurrent breast cancer after conservation therapy. Am J Surg.

[5] Fisher B, Anderson S, Redmond CK, Wolmark N, Wickerham DL, Cronin WM (1995). Reanalysis and results after 12 years of follow-up in a randomized clinical trial comparing total mastectomy with lumpectomy with or without irradiation in the treatment of breast cancer. N Engl J Med.

[6] Bartelink H, Horiot JC, Poortmans PM, Struikmans H, Van den Bogaert W, Fourquet A, Jager JJ, Hoogenraad WJ, Oei SB, Warlam-Rodenhuis CC, Pierart M, Collette L (2007). Impact of a higher radiation dose on local control and survival in breast-conserving therapy of early breast cancer: 10-year results of the randomized boost versus no boost EORTC 22881-10882 trial. J Clin Oncol.

[7] Athas WF, Adams-Cameron M, Hunt WC, Amir-Fazli A, Key CR (2000). Travel distance to radiation therapy and receipt of radiotherapy following breast-conserving surgery. J Natl Cancer Inst.

[8] Kirby RM, Basit A, Manimaran N (2008). Patient choice significantly affects mastectomy rates in the treatment of breast cancer. Int Semin Surg Oncol.

[9] Pezner RD, Tan MC, Clancy SL, Chen YJ, Joseph T, Vora NL (2013). Radiation therapy for breast cancer patients who undergo oncoplastic surgery: localization of the tumor bed for the local boost. Am J Clin Oncol.

[10] Malter W, Kirn V, Richters L, Fridrich C, Markiefka B, Bongartz R, Semrau R, Mallmann P, Kraemer S (2014). Intraoperative Boost Radiotherapy during Targeted Oncoplastic Breast Surgery: Overview and Single Center Experiences. Int J Breast Cancer.

[11] Schnitt SJ, Connolly JL, Harris JR, Hellman S, Cohen RB (1984). Pathologic predictors of early local recurrence in Stage I and II breast cancer treated by primary radiation therapy. Cancer.

[12] Veronesi U, Gatti G, Luini A, Intra M, Orecchia R, Borgen P, Zelefsky M, McCormick B, Sacchini V (2003). Intraoperative radiation therapy for breast cancer: technical notes. Breast J.

[13] Clark RM, Wilkinson RH, Mahoney LJ, Reid JG, MacDonald WD (1982). Breast cancer: a 21 year experience with conservative surgery and radiation. Int J Radiat Oncol Biol Phys.

[14] Veronesi U, Luini A, Del VM, Greco M, Galimberti V, Merson M, Rilke F, Sacchini V, Saccozzi R, Savio T, Zucali R, Zurrida S, Salvadori B (1993). Radiotherapy after breast-preserving surgery in women with localized cancer of the breast. N Engl J Med.

[15] Fisher ER, Anderson S, Redmond C, Fisher B (1992). Ipsilateral breast tumor recurrence and survival following lumpectomy and irradiation: pathological findings from NSABP protocol B-06. Semin Surg Oncol.

[16] Orecchia R, Ciocca M, Lazzari R, Garibaldi C, Leonardi MC, Luini A, Intra M, Gatti G, Veronesi P, Petit JI, Veronesi U (2003). Intraoperative radiation therapy with electrons (ELIOT) in early-stage breast cancer. Breast.

[17] Veronesi U, Orecchia R, Maisonneuve P, Viale G, Rotmensz N, Sangalli C, Luini A, Veronesi P, Galimberti V, Zurrida S, Leonardi MC, Lazzari R, Cattani F (2013). Intraoperative radiotherapy versus external radiotherapy for early breast cancer (ELIOT): a randomised controlled equivalence trial. Lancet Oncol.

[18] Vaidya JS, Wenz F, Bulsara M, Tobias JS, Joseph DJ, Keshtgar M, Flyger HL, Massarut S, Alvarado M, Saunders, Eiermann W, Metaxas M, Sperk E (2014). Risk-adapted targeted intraoperative radiotherapy versus whole-breast radiotherapy for breast cancer: 5-year results for local control and overall survival from the TARGIT-A randomised trial. Lancet.

[19] Esposito E, Anninga B, Harris S, Capasso I, D'Aiuto M, Rinaldo M, Douek M (2015). Intraoperative radiotherapy in early breast cancer. Br J Surg.

[20] Harris JR, Levene MB, Svensson G, Hellman S (1979). Analysis of cosmetic results following primary radiation therapy for stages I and II carcinoma of the breast. Int J Radiat Oncol Biol Phys.

[21] Whelan TJ, Pignol JP, Levine MN, Julian JA, MacKenzie R, Parpia S, Shelley W, Grimard L, Bowen J, Lukka H, Perera F, Fyles A, Schneider K (2010). Long-term results of hypofractionated radiation therapy for breast cancer. N Engl J Med.

[22] Bentzen SM, Agrawal RK, Aird EG, Barrett JM, Barrett-Lee PJ, Bentzen SM, Bliss JM, Brown J, Dewar JA, Dobbs HJ, Haviland JS, Hoskin PJ, Hopwood P (2008). The UK Standardisation of Breast Radiotherapy (START) Trial B of radiotherapy hypofractionation for treatment of early breast cancer: a randomised trial. Lancet.

[23] Bentzen SM, Agrawal RK, Aird EG, Barrett JM, Barrett-Lee PJ, Bliss JM, Brown J, Dewar JA, Dobbs HJ, Haviland JS, Hoskin PJ, Hopwood P, Lawton PA (2008). The UK Standardisation of Breast Radiotherapy (START) Trial A of radiotherapy hypofractionation for treatment of early breast cancer: a randomised trial. Lancet Oncol.

[24] Haviland JS, Owen JR, Dewar JA, Agrawal RK, Barrett J, Barrett-Lee PJ, Dobbs HJ, Hopwood P, Lawton PA, Magee BJ, Mills J, Simmons S, Sydenham MA (2013). The UK Standardisation of Breast Radiotherapy (START) trials of radiotherapy hypofractionation for treatment of early breast cancer: 10-year follow-up results of two randomised controlled trials. Lancet Oncol.

[25] Veronesi U, Orecchia R, Luini A, Gatti G, Intra M, Zurrida S, Ivaldi G, Tosi G, Ciocca M, Tosoni A, De Lucia F (2001). A preliminary report of intraoperative radiotherapy (IORT) in limited-stage breast cancers that are conservatively treated. Eur J Cancer.

[26] Kawamura M, Itoh Y, Sawaki M, Kikumori T, Tsunoda N, Kamomae T, Kubota S, Okada T, Nakahara R, Ito J, Hayashi H, Naganawa S (2015). A phase I/II trial of intraoperative breast radiotherapy in an Asian population: 5-year results of local control and cosmetic outcome. Radiat Oncol.

[27] Maluta S, Dall'Oglio S, Marciai N, Gabbani M, Franchini Z, Pietrarota P, Meliadò G, Guariglia S, Cavedon C (2012). Accelerated partial breast irradiation using only intraoperative electron radiation therapy in early stage breast cancer. Int J Radiat Oncol Biol Phys.

